# Supplementary material for: The archaeal protein SepF is essential for cell division in Haloferax volcanii
Source: Nat Commun. 2021 Jun 8;12:3469. doi: 10.1038/s41467-021-23686-9 (PMC8187382; doi:10.1038/s41467-021-23686-9)
Supplement: Supplementary file 1 — Supplementary Information [file 41467_2021_23686_MOESM1_ESM.pdf]

Supplementary Material to
The archaeal protein SepF is essential for cell division in *Haloferax volcanii*

Phillip Nußbaum [1], Maren Gerstner [1], Marie Dingethal [1], Celine Erb [1] and Sonja-Verena Albers [1] *

[1] Molecular Biology of Archaea, Institute of Biology II, Faculty of Biology, University of Freiburg, Germany

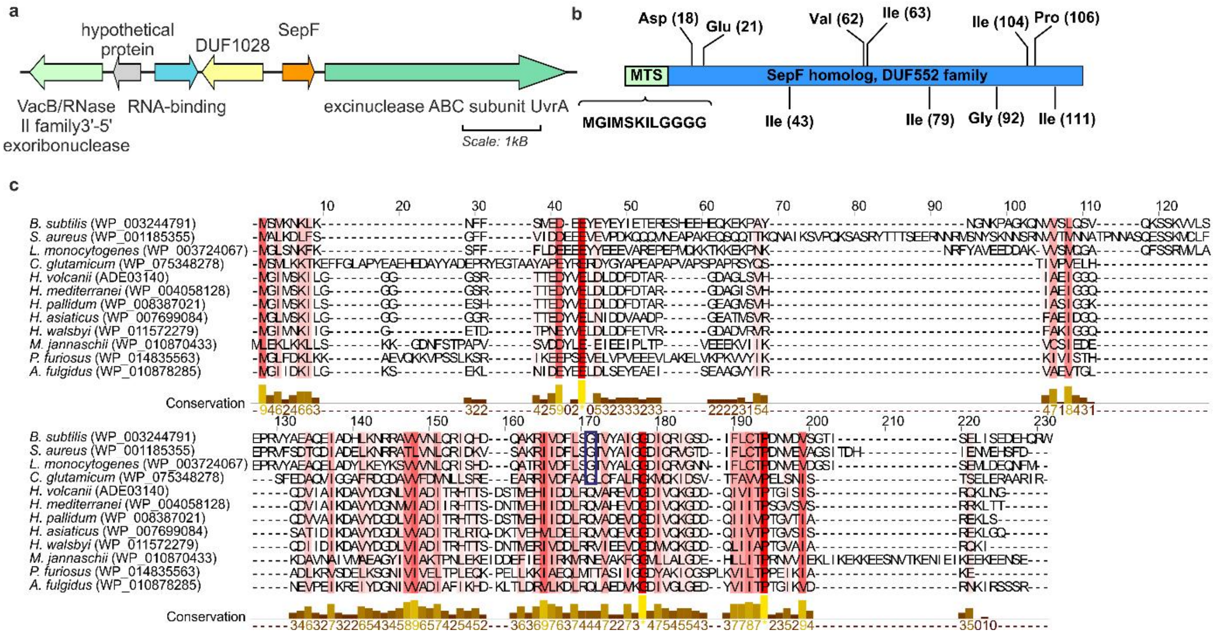

Supplementary Figure 1: Bioinformatical analysis of putative archaeal SepF proteins. a Genetic-neighborhood map of *hvo_0392*. Mapped are 6500 bp of the *hvo_0392* region. The UvrA protein is a DNA-interacting ATPase and part of the UvrABC repair system that recognizes DNA lesions (1). The closest up-stream gene of *hvo_0392* encodes for a protein with a domain of unknown function (DUF1028). According to the Pfam database (2) some proteins of this family are associated with peptidoglycan binding. The following up-stream genes encode for an RNA-binding protein, a hypothetical protein and a VacB/RNase family II 3'-5' exoribonuclease. **b** Schematic overview of the putative SepF protein from *H. volcanii*. The sequence of the membrane targeting sequence (MTS) (green) is indicated below and residues conserved between archaea and bacteria are highlighted. **c** Aligned protein sequences of bacterial SepFs with putative archaeal SepF proteins. Conserved residues are highlighted in a red color gradient from light red (conservation score of 6) to dark red (completely conserved). Residue G109 is indicated by a blue box. The conservation scores per site are indicated below the alignment.

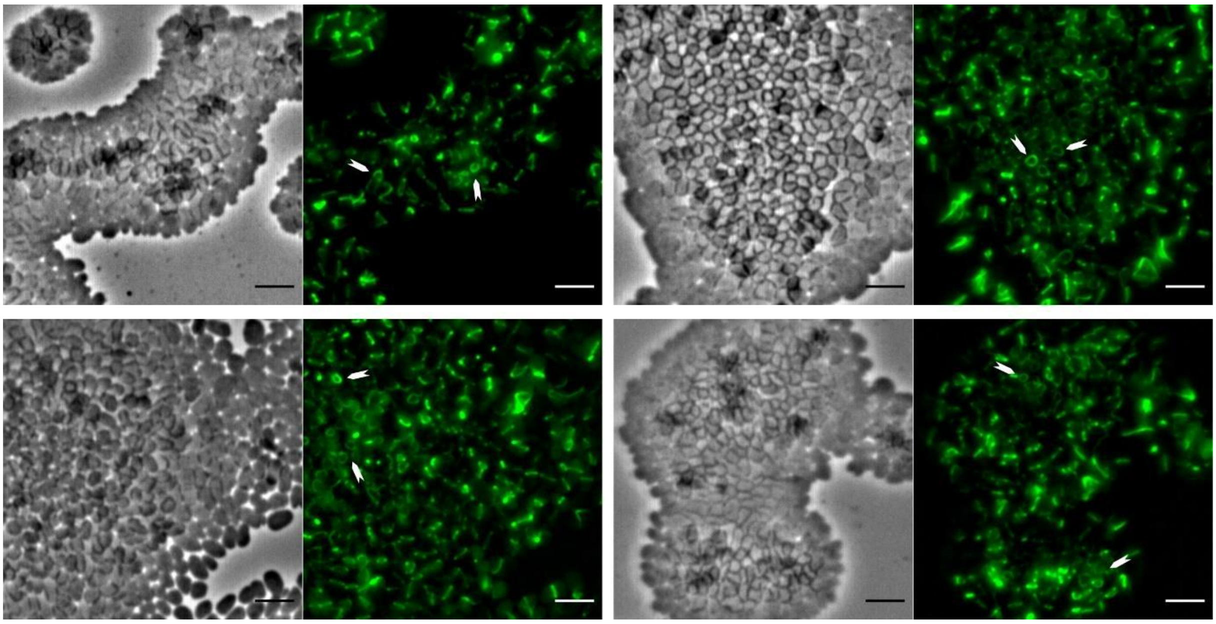

Supplementary Figure 2: SepF forms ring-like structures. Microscopy pictures of H26 expressing SepF-GFP from plasmid pSVA3942 grown into microcolonies on agarose pads with nutrients. In tilted cells formation of SepF-GFP into a ring like structures is observable indicated by white arrows. Images were chosen from two independent experiments. Scale bar: 4 µm.

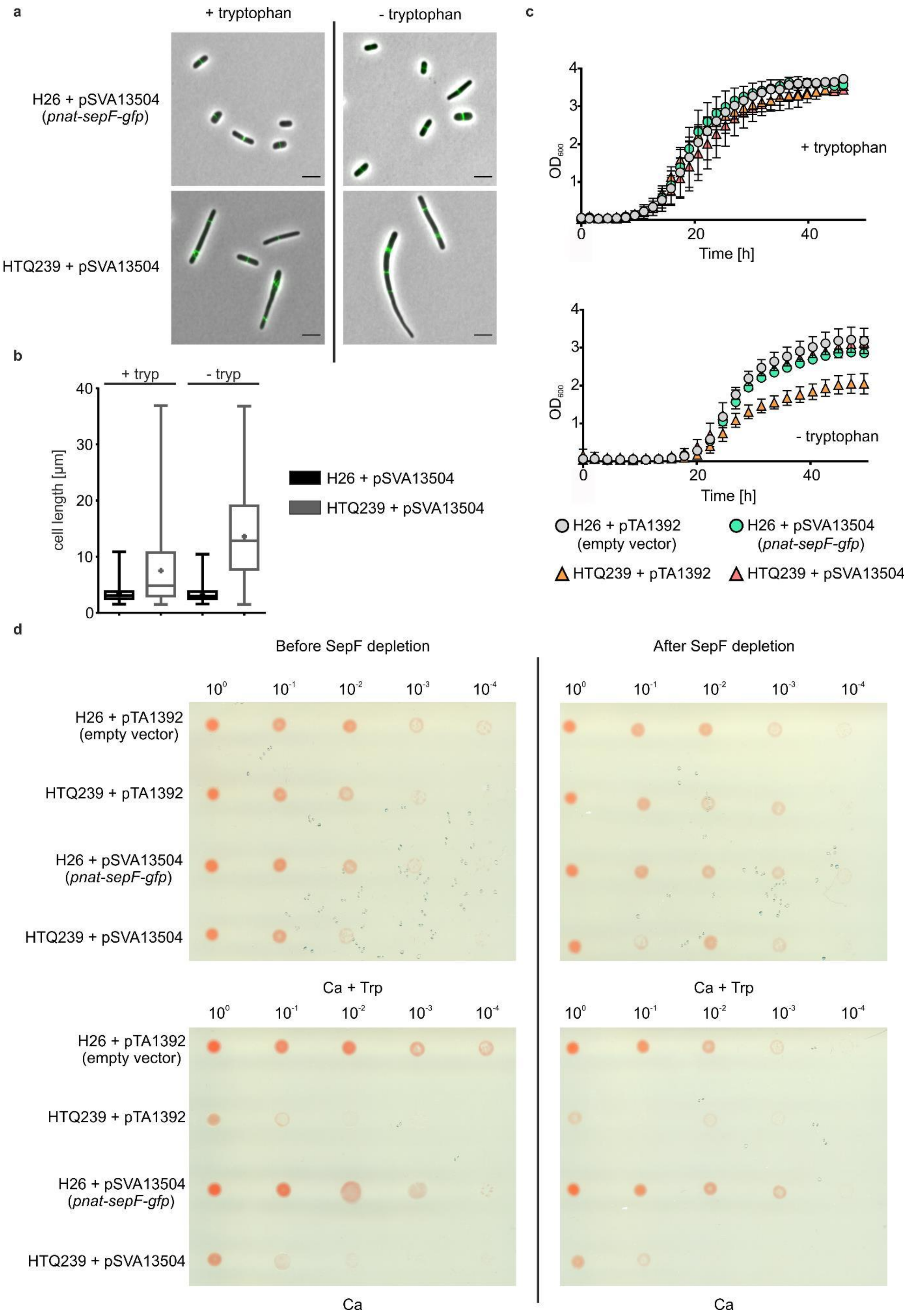
a
+ tryptophan
- tryptophan
H26 + pSVA13504
(pnat-sepF-gfp)
HTQ239 + pSVA13504
b
+ tryp
- tryp
cell length [µm]
H26 + pSVA13504
HTQ239 + pSVA13504
c
OD600
Time [h]
+ tryptophan
- tryptophan
H26 + pTA1392
(empty vector)
H26 + pSVA13504
(pnat-sepF-gfp)
HTQ239 + pTA1392
HTQ239 + pSVA13504
d
Before SepF depletion
After SepF depletion
10^0 10^-1 10^-2 10^-3 10^-4
H26 + pTA1392
(empty vector)
HTQ239 + pTA1392
H26 + pSVA13504
(pnat-sepF-gfp)
HTQ239 + pSVA13504
Ca + Trp
Ca

Supplementary Figure 3: Investigation of the functionality of the SepF-GFP phusion protein. a Fluorescence microscopy image of H26 and SepF-depletion strain HTQ239, both containing SepF-GFP expression plasmid pSVA13504 under SepF depleting (- tryptophan) and no depleting conditions (+tryptophan) (scale bar: 4µm). **b** Analysis of the cell length of the cells imaged in **a**. Data are shown as box plots, the minimum and maximum values are indicated by the whiskers, the median is indicated by a horizontal line and the mean by a +. Three biological replicates per strain were imaged and analyzed. **c** Growth curves of H26 and HTQ239 harboring the SepF-GFP expression plasmid or an empty plasmid control (pTA1391) in Cab-medium supplemented with tryptophan or without. For each strain three separate growth curves were acquired. Error bars indicate the standard deviation **d** Spot survival assay of H26 and HTQ239 containing the indicated plasmids. Left, cells were cultured under non SepF depletion conditions before they were either spotted on Ca plates containing tryptophan or on plates without tryptophan. Right, cells were grown under SepF depletion conditions prior to spotting on plates with or without tryptophan. Three biological replicates per strain were investigated.

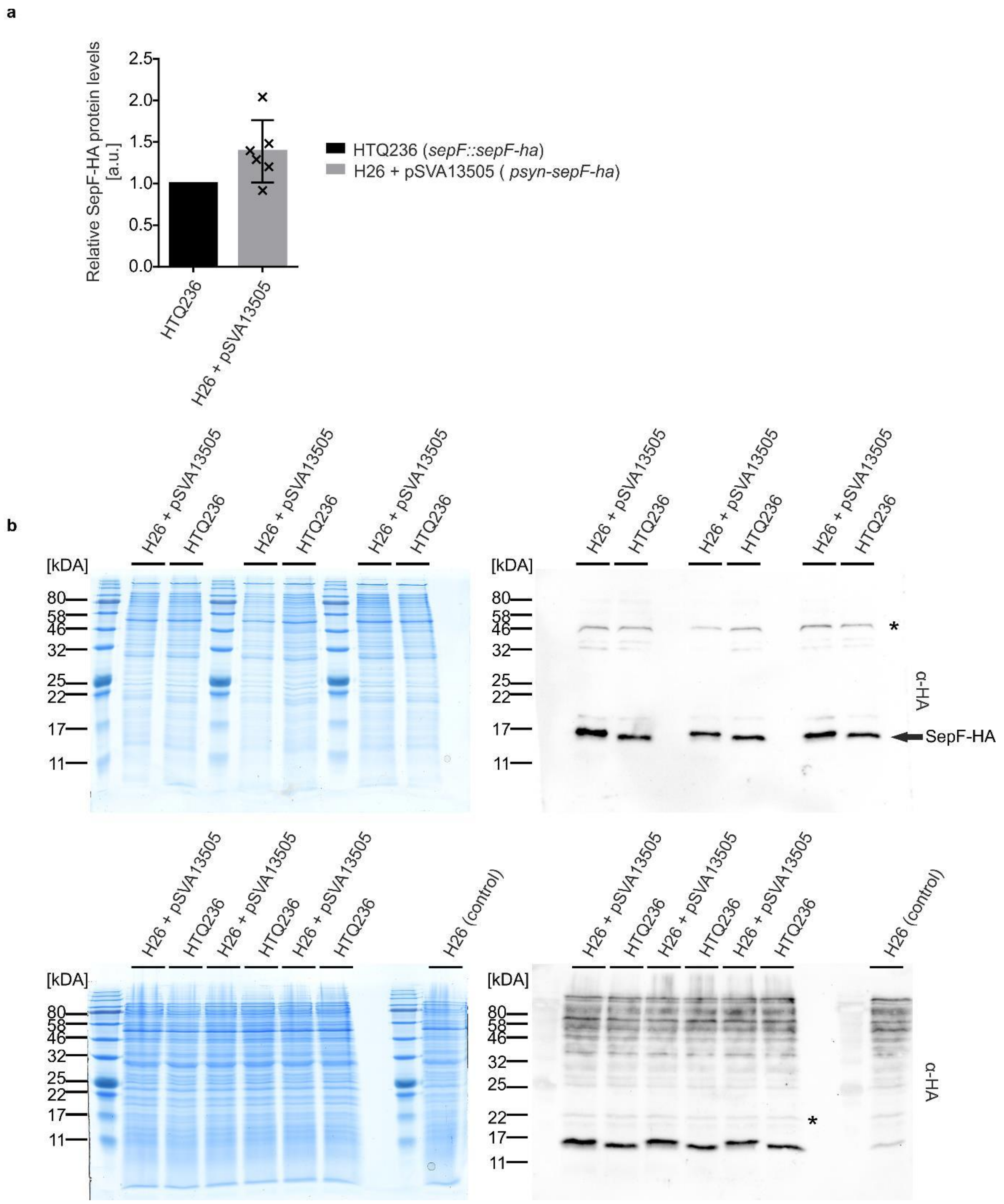

Supplementary Figure 4: Quantification of SepF overexpression under the control of a synthetic promotor. To assess the strength of the synthetic promotor (*psyn*) used for SepF overexpression *sepF* fused to an HA-taq was cloned under the control of *psyn* and the expression levels compared to strain HTQ236 (*sepF::sepF-ha*). **a** Relative protein levels of SepF-HA produced from the genome in HTQ236 and from plasmid in H26 + pSVA13505. Error bars indicate the standard deviation, the value of each replication is indicated by a x. **b** Loading controls and Western blots used for analysis. SepF-HA runs at a molecular weight of around 16 kDA, bands that were used for normalization on each blot are indicated by a *. In total six biological replicates per strain were analyzed.

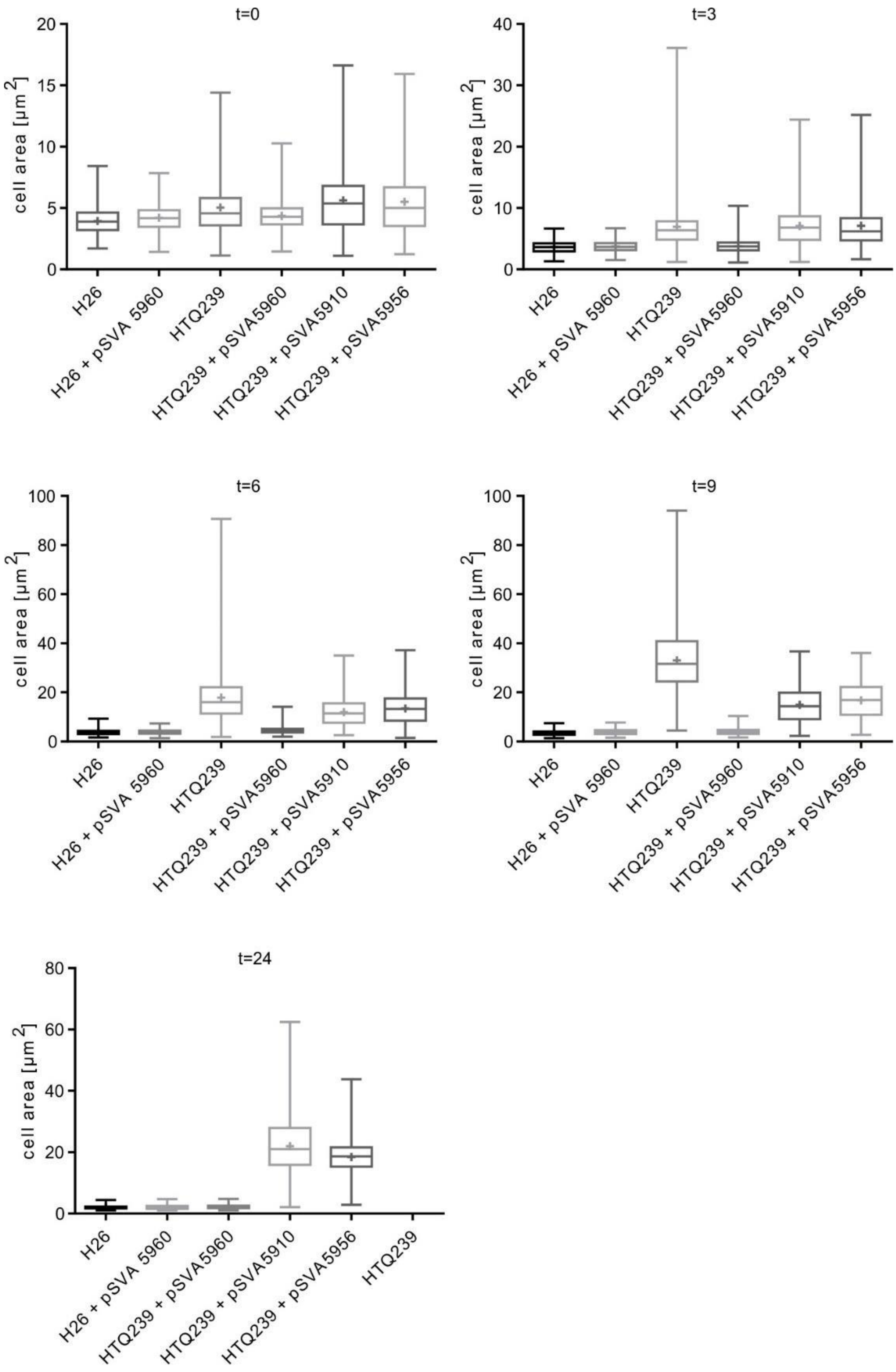

Supplementary Figure 5: Summary of all the cell areas measured in the indicated strains before and 3, 6, 9, 24 h after SepF depletion. Data are shown as box plots, the minimum and maximum values indicated by the whiskers, the median is indicated by a horizontal line and the mean by a +. The plots summarize the results from three independent experiments per strain and timepoint after depletion. Per timepoint and biological replicate > 700 cells per strain were analyzed.

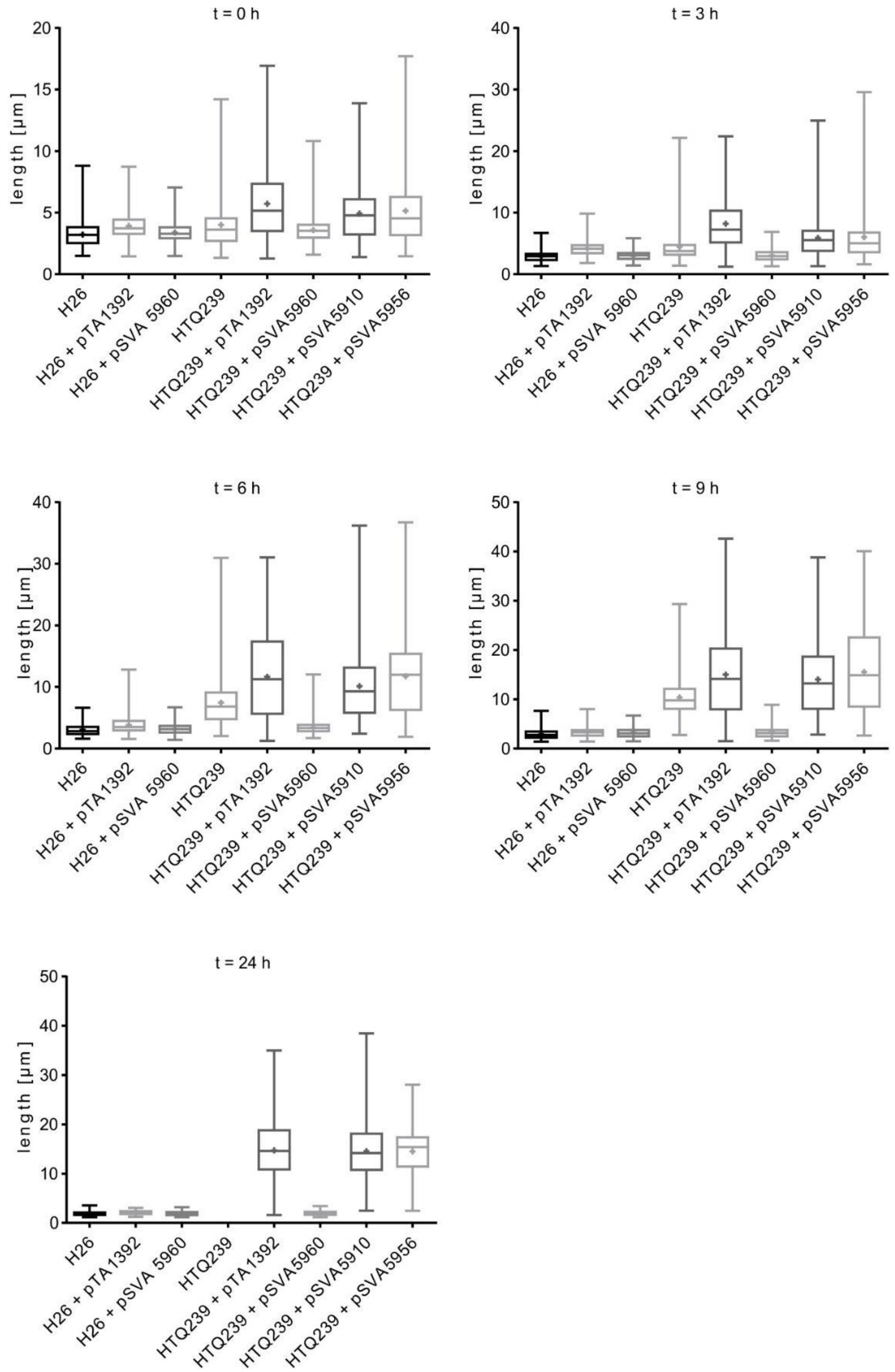

Supplementary Figure 6: Summary of all the cell lengths measured in the indicated strains before and 3, 6, 9, 24 h after SepF depletion. Data are shown as box plots, the minimum and maximum values indicated by the whiskers, the median is indicated by a horizontal line and the mean by a +. The plots summarize the results from three independent experiments per strain and timepoint after depletion. Per timepoint and biological replicate > 700 cells per strain were analyzed.

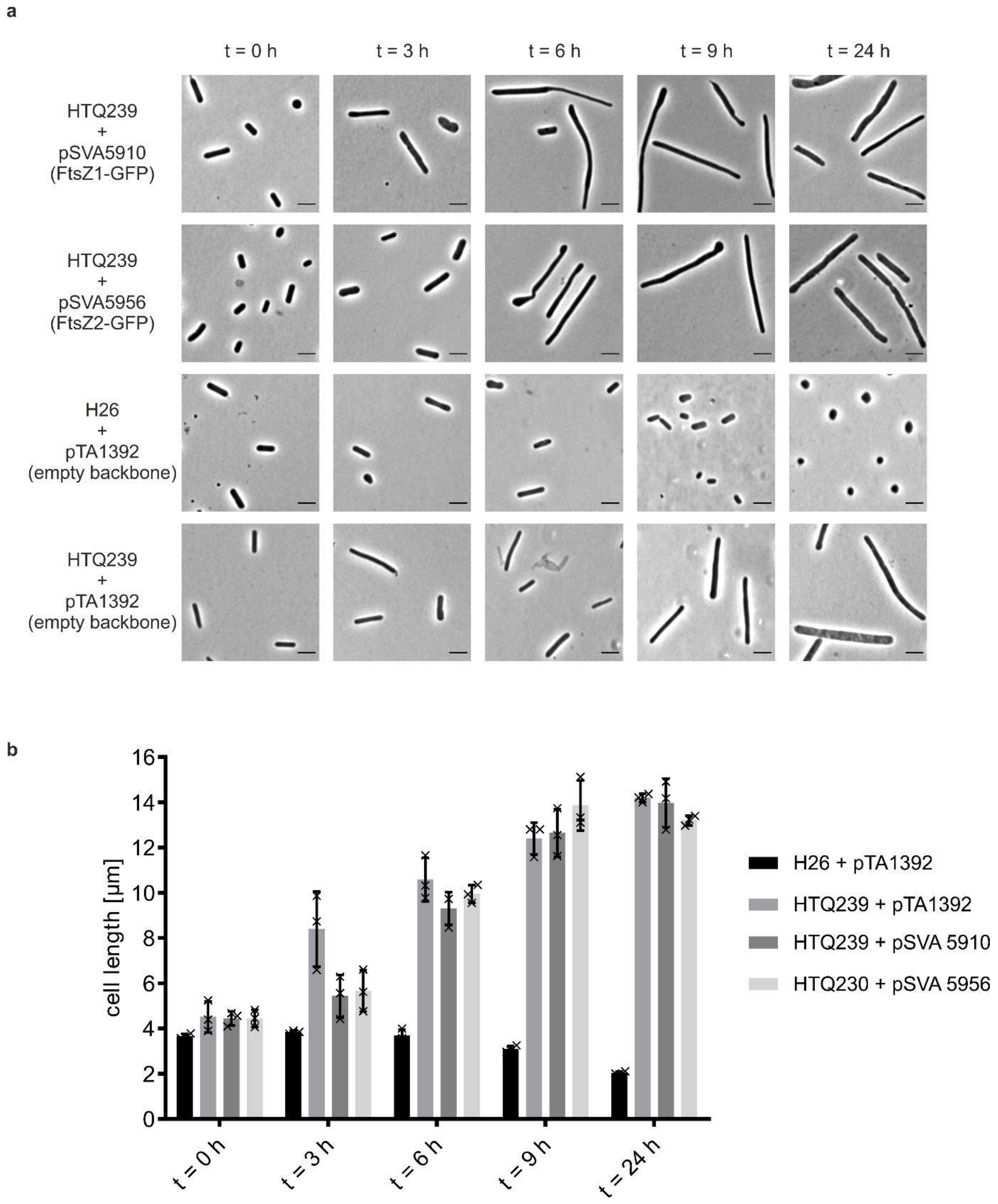

Supplementary Figure 7: Presence of a plasmid results into filamentation of HTQ239. a Microscopy pictures of HTQ239 containing either FtsZ1-GFP or FtsZ2-GFP expression plasmids and pictures of H26 and HTQ239 containing empty backbone plasmid pTA1392 at different time points after SepF depletion. Scale bars: 4 µm. **b** Mean cell length of strainHTQ239 + pSVA5910, HTQ239 + pSVA5956 H26 + pTA1392 and HTQ239 + pTA1392 0, 3, 6, 9 and 24 h after SepF depletion. Cell length was obtained from three independent experiments including the cell size of > 700 cells per strain and time point. Error bars indicate the standard deviation, the mean of each replication is indicated by a x.

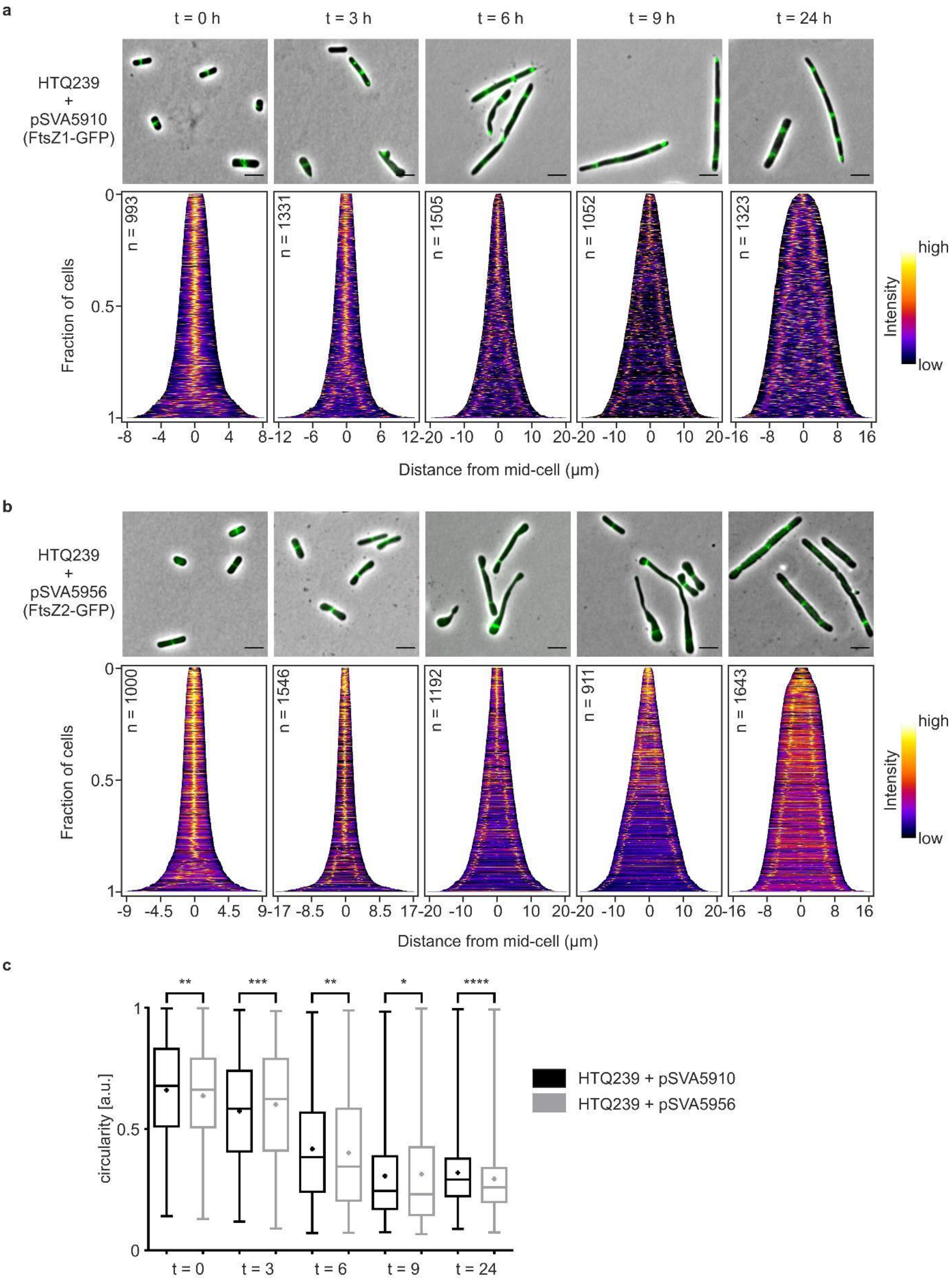

Supplementary Figure 8: Localization of FtsZ1 and FtsZ2 in SepF-depleted cells. a Fluorescence microscopy of HTQ239 cells additionally expressing *ftsZ1-gfp* under the control of its native promotor at different time points (0, 3, 6, 9 and 24 h) after sepF depletion. Over time the cells became filamentous and the number of FtsZ1 rings increased up to five rings

per cell. Lower row: Demographic analysis of the FtsZ1-GFP signal observed in HT239 at the indicated time points after SepF depletion. The number of cells analyzed per time point is indicated in the respective demograph. **b** Localization of FtsZ2-GFP by fluorescence microscopy in HTQ239 at different time points after SepF depletion. Cells became filamentous over time and the number of FtsZ2 rings partially increased up to three rings per cell. Lower row: Demographic analysis of the FtsZ2-GFP signal imaged in the SepF-depleted HTQ239 cells at different time points. The number of cells analyzed per time point is indicated in the respective demograph. The demographs summarize the results from three independent experiments per strain. Scale bars: 4 µml. **c** Circularity data are shown as box plots, the minimum and maximum values are indicated by the whiskers, the median is indicated by a horizontal line and the mean by a +. Statistical analysis of the circularity of both strains between different timepoints after SepF depletion were calculated by a one-tailed Mann-Whitney-Wilcoxon test (p-values per timepoint: t = 0: 0.0074 (**); t = 3: 0.0007 (***); t = 6: 0.0012 (**); t = 9: 0.0393 (*); t = 24: <0.0001 (****); confidence interval 95 %). The box plot summarizes the results from three independent experiments per strain and timepoint after depletion. Per timepoint and biological replicate > 700 cells per strain were analyzed.

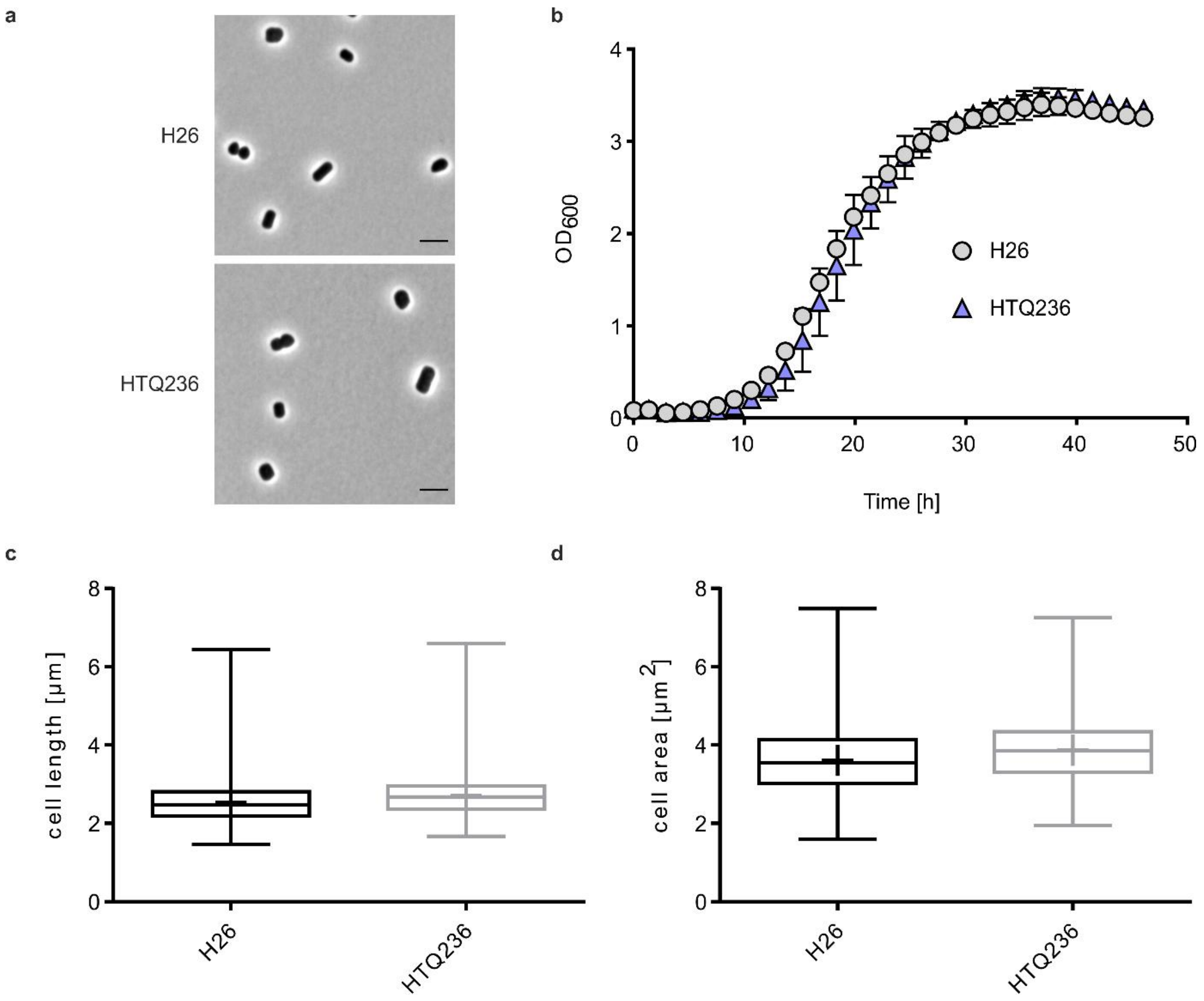

Supplementary Figure 9: Growth and cell shape comparison of strain H26 and HTQ236. **a** Phase contrast microscopy images of strain H26 and HTQ236 (*sepF::sepF-ha*) at an OD_{600} of 0.1 (scale bar: 4 µm). The experiment was repeated three times independently with similar results. **b** Growth curve of H26 and HTQ236 in Cab-medium supplemented with uracil. For each strain three growth curves were acquired. Error bars indicate the standard deviation. **c** Box plots of the cell length analysis of HTQ236 compared to H26. **d** Box plots of the cell area analysis of both strains. The minima and maxima of length and area are indicated by the whiskers, the medians are indicated by a horizontal line and the means by a +. For cell shape analysis three biological replicates per strain at an OD_{600} of 0.1 were analyzed.

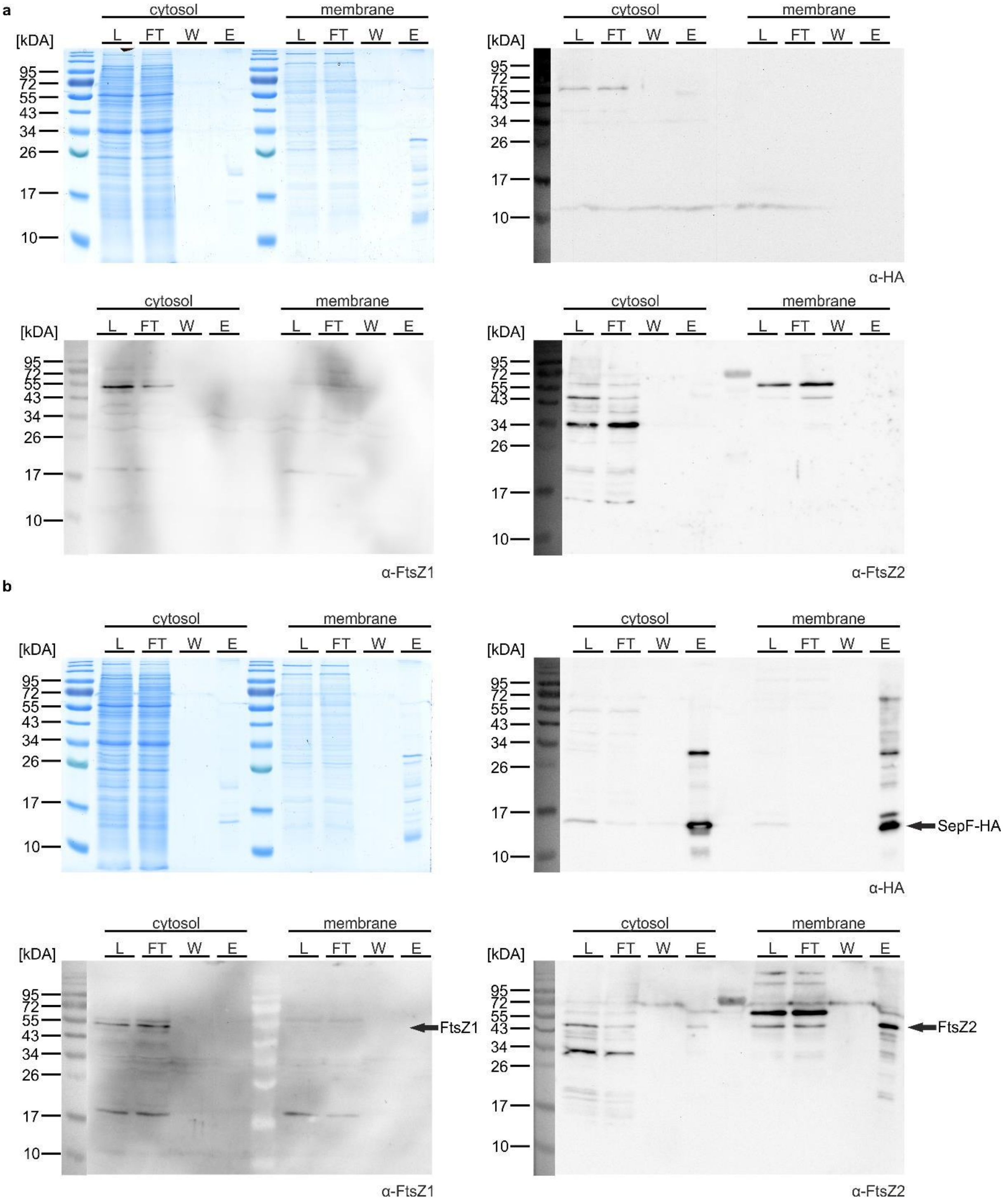

Supplementary Figure 10: Comprehensive gels and Western blots of SepF-HA co-immunoprecipitation. Gels and Western blots of the co-immunoprecipitation (co-IP) experiment using H26 **a** as control and strain HTQ236 **b** with a genomic *ha* tag on *sepF*. Cytosol and membrane fraction of both strains were separated and used for the co-IP experiment. Ten microliter of the load (L), flow through (FT), last wash (W) and elution (E) fraction were loaded on the gels. Loading controls were stained with Q-Stain (FastGene®) and the Western blots treated either with anti-HA, anti-FtsZ1 or anti-FtsZ2 antibodies respectively. The results come from one pulldown experiment.

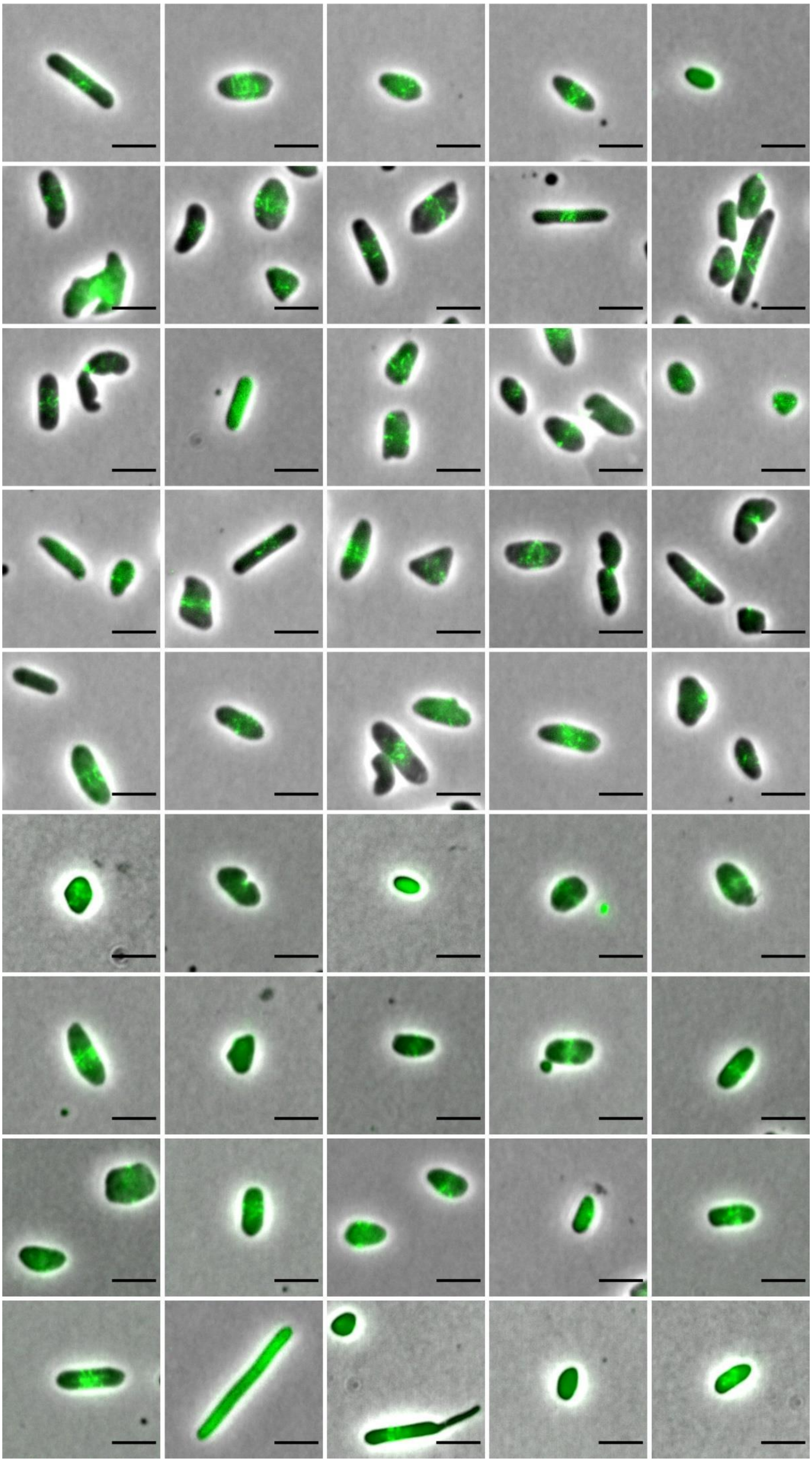

Supplementary Figure 11: Selection of less deformed *ΔftsZ1* cells expressing SepF-GFP. Although the cell shape of the shown cells is rather undisrupted, SepF-GFP is not organized into a distinct focus. Selections were taken from three independent biological replicates. Scale bars: 4 µm.

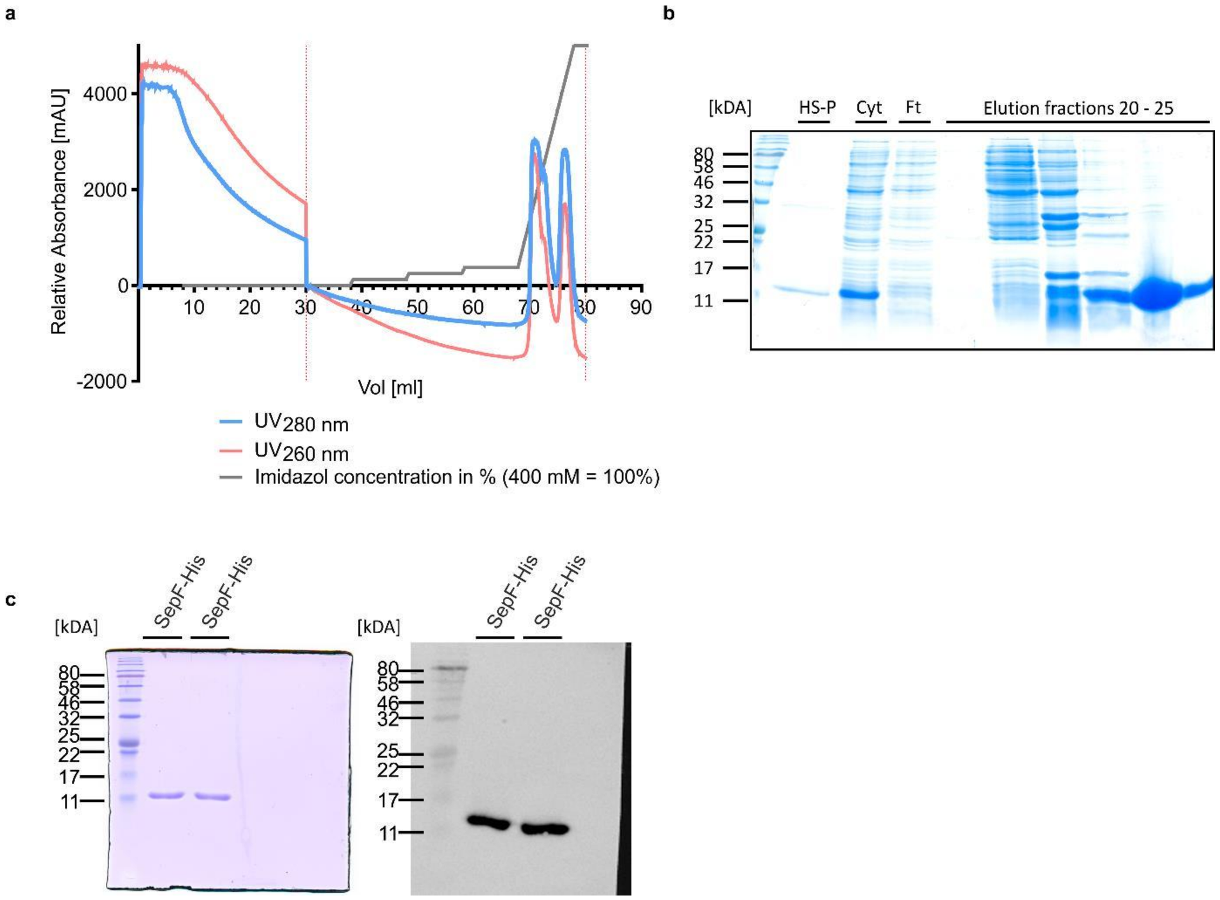

Supplementary Figure 12: SepF purification. a Chromatogram of the purification measured at 280 nm (blue) and 260 nm (red). The imidazole gradient is indicated by a grey line. **b** SDS-gel of the purification showing the pellet fraction of the high spin centrifugation step (HS-P), the cytosolic fraction (Cyt) used for purification the flow-through (Ft) and the elution fractions 20-25 (10 µl samples per pocket). The purification was repeated three times independently with similar results. **c** Whole Western blot image of SepF-His after size exclusion chromatography.

Supplementary Table 1: Strains used in this study

Strain Name	Background strain	Genotype	Source/reference
H. volcanii			
H26	-	Δ*pyrE2*	(3)
H98	-	Δ*pyrE2* Δ*hdrB*	(3)
HTQ236	H26	Δ*pyrE2 hvo_0392*::[*hvo_0392-ha*]	This study
HTQ239	H98	Δ*pyrE2* Δ*hdrB hvo_0392*::[*p.tnaA-hvo_0392-hdrB*$^{+}$]	This study
ID76	H98	Δ*pyrE2* Δ*hdrB p.fdx-hdrB* Δ*ftsZ1*	(4)
ID77	H98	Δ*pyrE2* Δ*hdrBp.fdx-hdrB* Δ*ftsZ2*	(4)
ID112	ID77	Δ*pyrE2* Δ*hdrB p.fdx-hdrB* Δ*ftsZ1* Δ*ftsZ2*	(4)
E. coli			
10-beta Competent Cells "TOP10"	-	Δ*(ara-leu) 7697 araD139 fhuA* Δ*lacX74 galK16 galE15 e14-* ϕ*80*d*lacZ*Δ*M15 recA1 relA1 endA1 nupG rpsL* (Str^{R}) *rph spoT1* Δ*(mrr-hsdRMS-mcrBC)*	New England Biolabs
dam^{-}/*dcm*$^{-}$ Competent	-	*ara-14 leuB6 fhuA31 lacY1 tsx78 glnV44 galK2 galT22 mcrA dcm-6 hisG4 rfbD1 R(zgb210::Tn10)* Tet^{S} *endA1 rspL136* (Str^{R}) *dam13::Tn9* (Cam^{R}) *xylA-5 mtl-1 thi-1 mcrB1 hsdR2*	New England Biolabs
Rosetta™(DE3) Competent Cells	-	F^{-} *ompT hsdS*$_{B}$(r_B^- m_B^-) *gal dcm* (DE3) pRARE (Cam^{R})	Novagen

Supplementary Table 2: Plasmids used in this study

Plasmids	Description	Primers used	Enzymes used	Source/reference
pTA131	Integrative plasmid with a *pyrE2* selection marker for knock-outs in *H. volcanii* (Amp^{r})	-	-	(3)
pIDJL-40	Plasmid for the expression of proteins with a C-terminal GFP-tag and a *pyrE2, hdrB* selection markers (Amp^{r})	-	-	(5)
pIDJL-114	Plasmid for the expression of proteins with a C-terminal mCherry-tag and *pyrE2, hdrB* selection markers (Amp^{r})			Iain Duggin
pTA1392	Plasmid for the expression of proteins in *H. volcanii* under control of *p.tnaA* and *pyrE2, hdrB* selection markers			(6)

	(Amp^r)			
pTA1369	Integrative plasmid with a *pyrE2* and *hdrB* selection marker containing the *p.tnaA1* promotor to generate a tryptophan inducible allele of a gene (Amp^r)	-	-	(7)
pTA1992	Plasmid for the constitutive expression of proteins in *H. volcanii* under control of as synthetic promotor (*pSyn)* with *pyrE2, hdrB* selection markers (Amp^r)			(7)
p7XC3H	FX cloning *E. coli* expression vector with T7 promoter and C-terminal 3C protease cleavage site and 10x His-tag (Kan^r)	-	-	(8)
p7XC3S	FX cloning *E. coli* expression vector with T7 promoter and C-terminal 3C protease cleavage site and Strep-tag (Kan^r)			(8)
pSVA3922	Plasmid for the expression of proteins with a N-terminal GFP-tag and *pyrE2, hdrB* selection markers (Amp^r)			(9)
pSVA3933	integrative plasmid with a *pyrE2* selection marker to generate a *hvo_0392* knock-out mutant (Amp^r)	8025, 8026; 8027, 8028	KpnI, BamHI; BamHI, XbaI	This study
pSVA3943	Plasmid to express two proteins, one with a c-terminal GFP tag and on with a c-terminal mCherry tag under the control of *p.tnaA. pyrE2* selection marker based on pTA1392 (Amp^r)	7010 7011<br><br>8062, 8063	NheI, EcoRI<br><br>BamHI, NotI	This study
pSVA3942	Tryptophan inducible SepF-GFP expression plasmid with a *pyrE2* selection marker (Amp^r)	8066, 8067	NdeI, BamHI	This study
pSVA3947	Integrative plasmid with a *pyrE2* selection marker to integrate an endogenous HA-tag at the C-terminus of SepF (Amp^r)	8068. 8069; 8070, 8071	KpnI, XbaI	This study
pSVA3951	Tryptophan inducible GFP-SepF expression plasmid	8083, 8084	NheI, BamHI	This study

	with a *pyrE2* selection marker<br>(Amp^r)			
pSVA3954	Integrative plasmid for the exchange of *hvo_0392* native promoter<br>(Amp^r)	8096;<br>8097	NdeI, EcoRI;<br>BglII, BamHI	This study
pSVA3968	Tryptophan inducible SepF-GFP, FtsZ1-mCherry double expression plasmid with a *pyrE2* selection marker<br>(Amp^r)	8092;<br>8093<br><br>8096,<br>8802	EcoRI, BamHI<br>(for FtsZ1)<br><br>NheI, NdeI<br>(for SepF)	This study
pSVA3969	Plasmid for expression of SepF with a C-terminal 10 x His-tag in *E. coli* based on p7XC3H<br>(Kan^r)	8803,<br>8804	SapI	This study
pSVA3970	Plasmid for expression of FtsZ1 with a C-terminal 10 x His-tag in *E. coli* based on p7XC3H<br>(Kan^r)	8805,<br>8806	SapI	This study
pSVA5910	Plasmid for expression of FtsZ1-GFP under the control of its native promotor<br>(Amp^r)	9703,<br>9704	ApaI,<br>BamHI	This study
pSVA5912	Plasmid for expression of FtsZ2 with a C-terminal Strep-tag in *E. coli* based on p7XC3S<br>(Kan^r)	9705,<br>9706	SapI	This study
pSVA5953	Tryptophan inducible SepF-GFP, FtsZ2-mCherry double expression plasmid with a *pyrE2* selection marker. Based on pSVA3968<br>(Amp^r)	9707,<br>9788	EcoRI,<br>BamHI	This study
pSVA5954	Tryptophan inducible $SepF_{\Delta MTS}$-GFP expression plasmid with a *pyrE2* selection marker<br>(Amp^r)	9789,<br>8067	NdeI,<br>BamHI	This study
pSVA5955	Tryptophan inducible MTS_{SepF}-GFP expression plasmid with a *pyrE2* selection marker<br>(Amp^r)	11013,<br>11014	NdeI, NotI	This study
pSVA5956	Plasmid for expression of FtsZ2-GFP under the control of its native promotor with a *pyrE2* selection marker<br>(Amp^r)	9793,<br>9794	ApaI/BamHI	This study
pSVA5960	Plasmid for constitutive expression of SepF under	8867,<br>8024	NcoI, NheI<br>(insert);	This study

	the control of a synthetic promotor with a *pyrE2* selection marker (Amp^r)		PciI, NheI (plasmid)	
pSVA13504	Expression plasmid for SepF-GFP expression under control of its native promotor with a *pyrE2* selection marker (Amp^r)	11080, 8067	ApaI, BamHI	This study
pSVA13505	Plasmid for constitutive expression of SepF-HA under the control of a synthetic promotor with a *pyrE2* selection marker (Amp^r)	11081, 11083	PCR on pSVA5960	This study

Supplementary Table 3: Primers used in this study

Primer Number/Name	Sequence 5'→3'	Description
7010	CGGCTAGCATGAGTAAAGGAGAAGAACTTTTCACTGGAGTTGTCCCAATTCTTGTTG	Forward primer for the amplification of *gfp* with a NheI restriction site (cloned in pTA1392)
7011	GGAATTCTCACTTCTCGAACTGCGGGTGCGACCATTTGTATAGTTCATCCATGCCATG	Reverse primer for the amplification of *gfp* with an EcoRI restriction site (cloned in pTA1392)
8024	TCTAGCTAGCGCCGTTGAGCTTCTGC	Reverse primer for the amplification of *hvo_0392* with NheI restriction site (cloned in pIDJL-40 and pTA1992)
8025	GATAGGTACCAGCGAACGTCGCCTCGGAAC	Forward primer for the amplification of the up-stream region of *hvo_0392* with KpnI restriction site (cloned in pTA131)
8026	GTAGGATCCAGGACGTAGAGAGGCCACAAC	Reverse primer for the amplification of the up-stream region of *hvo_0392* with BamHI restriction site (cloned in pTA131)
8027	GTAGGATCCCGACGGCGGACGACACCC	Forward primer for the amplification of the down-stream region of *hvo_0392* with BamHI restriction site (cloned in pTA131)
8028	GTACTCTAGAATGTCCTGTGCGGACTGCTC	Reverse primer for the amplification of the down-stream region of *hvo_0392* with XbaI restriction site (cloned in pTA131)
8062	CGGGATCCCCAATGCATGTGAGCAAGGGCGAGGAGGATAAC	Forward primer for the amplification of *mcherry* with a BamHI and NsiI restriction site (cloned in pTA1392)
8063	ATAAGAATGCGGCCGCTTACTTGTACAGCTCGTCCATGCC	Reverse primer for the amplification of *mcherry* with a NotI restriction site (cloned in pTA1392)

8066	GGAATTCCATATGGGTATCATGAGTAAGATTCTCG	Forward primer for the amplification of *hvo_0392* with NdeI restriction site (cloned in pIDJL-40)
8067	CTAGGATCCGCCGTTGAGCTTCTGCC	Reverse primer for the amplification of *hvo_0392* with BamHI restriction site (cloned in pIDJL-40 and pSVA5954)
8068	GGGGTACCGACGCCCGTTGGAACCGCCGACT	Forward primer for the amplification of ~500 Bp up-stream region of the *sepF* stop codon with a KpnI restriction site
8069	TCACGCGTAGTCCGGGACGTCGTACGGGTAGCTGCCGCCGTTGAGCTTCTGCC	Reverse primer for the amplification of ~500 Bp up-stream region of the *sepF* stop codon containing the coding sequence for a HA-tag + stoop codon
8070	GGCAGCTACCCGTACGACGTCCCGGACTACGCGTGACGACGGCGGACGACACC	Forward primer for the amplification of ~500 Bp down-stream region of the *sepF* stop codon containing the coding sequence for a HA-tag + a stop codon (the HA tag sequence is complementary to the on in primer 8069)
8071	TGCTCTAGAATGTCCTGTGCGGACTGCTC	Reverse primer for the amplification of ~500 Bp down-stream region of the *sepF* stop codon with a XbaI restriction site
8055	GCGCGTCGATGAGTCGTTTC	Forward primer to screen for successful HA-tag incorporation
8083	TCTAGCTAGCATCATGAGTAAGATTCTCGGTGGTG	Forward primer for the amplification of *hvo_0392* with NheI restriction site (cloned in pSVA3922)
8084	CTAGGATCCTCAGCCGTTGAGCTTCTG	Reverse primer for the amplification of *hvo_0392* with BamHI restriction site (cloned in pSVA3922)
8087	GTAGTCCGGGACGTCGTACGG	Reverse primer binding the HA-DNA sequence to check for successful tag incorporation in HTQ236
8092	CCGGAATTCATGGACTCTATCGTCGGCGAC	Forward primer for the amplification of *ftsZ1* with EcoRI restriction site (cloned in pSVA3943)
8093	CGCGGATCCCTCGACGTAGTCGATGTCTTC	Reverse primer for the amplification of *ftsZ1* with BamHI restriction site (cloned in pSVA3943)
8096	GGAATTCCATATGGGTATCATGAGTAAGATTCTCGG	Forward primer for the amplification of *hvo_0392* with NdeI restriction site (cloned in pTA1369 and pSVA3943)
8097	CCGGAATTCTCAGTGGTGGTGGTGGTGGTGGCCGTTGAGCTTCTGCCGC	Revers primer for the amplification of *hvo_0392* with a NdeI restriction site and 6 x His coding sequence (cloned in pTA1369)
8802	TCTAGCTAGCGCCGTTGAGCTTCTGC	Reverse primer for the amplification of *hvo_0392* with a NheI restriction site (cloned in pSVA3943)
8803	ATATATGCTCTTCTAGTGGTATCATGAGTAAGATTCTCGGTGGT	Forward primer for the amplification of *hvo_0392* with SapI restriction site (cloned in p7XC3H)

8804	CCAAGTGCTCTTCATGCGCC GTTGAGCTTCTGCCGCGAGA TAGA	Reverse primer for the amplification of *hvo_0392* with SapI restriction site (cloned in p7XC3H)
8805	ATATATGCTCTTCTAGTGACT CTATCGTCGGCGACGCAATT GAC	Forward primer for the amplification of *ftsZ1* with SapI restriction site (cloned in p7XC3H)
8806	TATATAGCTCTTCATGCTTCG ACGTAGTCAATGTCTTCGAGA CG	Reverse primer for the amplification of *ftsZ1* with SapI restriction site (cloned in p7XC3H)
8817	TCGTGGCGAGAACGGAACAG	Forward primer binding *p.tnaA* for colony PCR on HTQ239
8818	GGGCAGACGAACACGATGAC	Reverse primer binding *hvo_0392* for colony PCR on HTQ239
8867	CATGCCATGGGTATCATGAG TAAGATTCTCGG	Forward primer for the amplification of *hvo_0392* with NcoI restriction site (cloned in pTA1992)
9703	TTCGGGCCCAAGCTCTCGGC CGGGCAGTCC	Forward primer for the amplification of *ftsZ1* with its promotor region and an ApaI restriction site (cloned in pIDJL-40)
9704	CGCGGATCCCTCGACGTAGT CGATGTCTTC	Reverse primer for the amplification of *ftsZ1* with BamHI restriction site (cloned in pIDJL-40)
9705	ATATATGCTCTTCTAGTCAGG ATATTGTTCGCGAGGCGATG GAA	Forward primer for the amplification of *ftsZ2* with SapI restriction site (cloned in p7XC3S)
9706	TATATAGCTCTTCATGCCCGA ATGACGTCGAGACCGTTGTT CTT	Reverse primer for the amplification of *ftsZ2* with SapI restriction site (cloned in p7XC3S)
9707	CCGGAATTCATGCAGGATAT CGTTCGCGAG	Forward primer for the amplification of *ftsZ2* with EcoRI restriction site (cloned in pSVA3943)
9788	CCAATGCATCCGGATGACGT CGAGACC	Reverse primer for the amplification of *ftsZ2* with NsiI restriction site (cloned in pSVA3943)
9789	GGAATTCCATATGGGTGGTG GTTCCCGAAC	Forward primer for the amplification of truncated *hvo_0392* ($SepF_{\Delta MTS}$) with NdeI restriction site (cloned in pIDJL-40)
9793	TTCGGGCCCTTTCGGGTGTA AGACACTCG	Forward primer for the amplification of *ftsZ2* with its promotor region and an ApaI restriction site (cloned in pIDJL-40)
9794	CGCGGATCCCCGGATGACGT CGAGACC	Reverse primer for the amplification of *ftsZ2* with BamHI restriction site (cloned in pIDJL-40)
11013	ATTGCGCATATG*GGTATCATG AGTAAGATTCTCGGTGGTGG TGGT*GGATCCATGAGTAAAG GAGAAGAAC	Forward primer for the amplification of gfp containing the gene *sequence for the MTS of SepF* and NdeI restriction site (cloned in pTA1392)

11014	ATAAGAATGCGGCCGCTTATTTGTATAGTTCATCCATGCCATG	Reverse primer for the amplification of *gfp* with a NotI restriction site (cloned in pTA1392)
11080	TTCGGGCCCTGAAGGTCATCGTGTTCGTC	Forward primer for the amplification of *sepF* with its native promotor with ApaI restriction site (cloned in pIDJL-40)
11081	GGCAGCTACCCGTACGACGTCCCGGACTACGCGTGATGAGATATCGAATTCCTGC	Forward primer for the amplification of pSVA5960 introducing a C-terminal HA tag on SepF
11083	GCCGTTGAGCTTCTGCCGCG	Reverse primer for the amplification of pSVA5960

Supplementary Table 4: MS results from pulldown experiments. Identified proteins in the pulldown fractions of whole cell lysate from H26 and HTQ236 are shown. FtsZ2 exclusively found in the pulldown fraction of HTQ236 is highlighted (yellow).

Protein IDs (Uniprot)	Protein names	Peptide counts (unique)	Sequence coverage [%]	PEP	Intensity Sample from 1 (H26)	Intensity Sample from 2 (HTQ236)
D4GP60	Cobalt-factor-III C17-methyltransferase	1	5.4	2.13E-22	413500	235420
D4GP61	Cobalamin biosynthesis protein CbiG	1	4	0.0079128	44590	0
D4GP63	Cobalt-factor-II C20-methyltransferase	1	7.7	7.08E-05	144240	255540
D4GP64	Probable cobalt-precorrin-6B C(15)-methyltransferase (decarboxylating)	5	39.1	3.25E-59	951400	728940
D4GQU5	Class II aldolase (Homolog to L-fuculose-phosphate aldolase)	1	4.3	1.20E-10	134800	0
D4GRF0	Cobyrinate a,c-diamide synthase	3	10.9	5.62E-20	261230	628040
D4GSH7	Cell division protein FtsZ2	13	55.8	0	0	22549000
D4GSW9	Uridylate kinase	1	7.6	3.29E-39	141920	146280
D4GT59	Succinate--CoA ligase [ADP-forming] subunit alpha	2	9.3	3.63E-12	2417300	0
D4GTI4	Aconitate hydratase	7	18.7	1.66E-122	381180	1842800
D4GTJ3	PRC domain protein	2	37	5.28E-26	0	866450
D4GTX8	50S ribosomal protein L19e	5	33.1	3.71E-30	1559400	1037100
D4GTY4	30S ribosomal protein S4e	3	17	7.60E-45	198960	0
D4GTY7	30S ribosomal protein S17	2	23.7	2.37E-86	869560	392600
D4GU54;D4GZ05	GalE family epimerase/dehydratase	4;1	19.6	1.87E-243	620070	786560
D4GU72	Low-salt glycan biosynthesis protein Agl12	4	26.5	1.70E-76	588470	0
D4GUM1	Beta-lactamase domain protein	2	4.2	2.03E-19	87886	0
D4GW49	Ribonuclease J	3	9.6	1.80E-68	412660	443750
D4GWR9	Putative glycosyltransferase, type 1	1	4	0.00056575	171630	0
D4GXC7	UPF0173 metal-dependent hydrolase HVO_1290	1	6.3	7.88E-17	454890	262670
D4GYD4	D-gluconate dehydratase	1	6.8	8.28E-06	0	143940
D4GYN6	Ornithine carbamoyltransferase	2	11	1.07E-93	90063	141780
D4GYP0	Putative [LysW]-L-2-aminoadipate/[LysW]-	5	26.2	1.20E-131	1070500	2207500

	L-glutamate phosphate reductase					
D4GYQ7	ABC-type transport system periplasmic substrate-binding protein (Probable substrate dipeptide/oligopeptide)	2	5.9	1.16E-15	260690	143520
D4GZY6	Elongation factor 1-alpha	5	22.6	1.03E-148	517430	2306600
D4GZY7	30S ribosomal protein S10	2	22.5	3.51E-08	0	181910
D4H018	Putative SepF protein	6	80.5	7.29E-274	246670	74749000
D4H036	PRC domain protein	3	42.3	2.74E-27	0	6214600
O30560	Thermosome subunit 2	3	11.5	8.73E-30	370730	0
O30561	Thermosome subunit 1	3	9.8	1.68E-126	291550	0
Q48328	DNA repair and recombination protein RadA	4	15.7	5.80E-21	0	271570
Q48333	V-type ATP synthase beta chain	12	38.2	2.74E-130	2810300	772780

Supplementary References

1. Grossman,L. and Yeung,A.T. (1990) The UvrABC endonuclease system of Escherichia coli--a view from Baltimore. *Mutat. Res.*, **236**, 213–221.
2. El-Gebali,S., Mistry,J., Bateman,A., Eddy,S.R., Luciani,A., Potter,S.C., Qureshi,M., Richardson,L.J., Salazar,G.A., Smart,A., *et al.* (2019) The Pfam protein families database in 2019. *Nucleic Acids Res.*, **47**, D427–D432.
3. Allers,T., Ngo,H.-P., Mevarech,M. and Lloyd,R.G. (2004) Development of additional selectable markers for the halophilic archaeon *Haloferax volcanii* based on the *leuB* and *trpA* genes. *Appl. Environ. Microbiol.*, **70**, 943–53.
4. Liao,Y., Ithurbide,S., Löwe,J. and Duggin,I.G. (2020) Two FtsZ proteins orchestrate archaeal cell division through distinct functions in ring assembly and constriction. *bioRxiv*, 10.1101/2020.06.04.133736.
5. Duggin,I.G., Aylett,C.H.S., Walsh,J.C., Michie,K.A., Wang,Q., Turnbull,L., Dawson,E.M., Harry,E.J., Whitchurch,C.B., Amos,L.A., *et al.* (2015) CetZ tubulin-like proteins control archaeal cell shape. *Nature*, **519**, 362–365.
6. Gamble-Milner,R. (2016) Genetic analysis of the Hel308 helicase in the archaeon Haloferax volcanii. *PhD thesis, Univ. Nottingham*.
7. Braun,F., Thomalla,L., van der Does,C., Quax,T.E.F., Allers,T., Kaever,V. and Albers,S.-V. (2019) Cyclic nucleotides in archaea: Cyclic di-AMP in the archaeon Haloferax volcanii and its putative role. *Microbiologyopen*, **8**, 1–23.
8. Geertsma,E.R. and Dutzler,R. (2011) A versatile and efficient high-throughput cloning tool for structural biology. *Biochemistry*, **50**, 3272–3278.

9. Nußbaum,P., Ithurbide,S., Walsh,J.C., Patro,M., Delpech,F., Rodriguez-Franco,M., Curmi,P.M.G., Duggin,I.G., Quax,T.E.F. and Albers,S.V. (2020) An Oscillating MinD Protein Determines the Cellular Positioning of the Motility Machinery in Archaea. *Curr. Biol.*, **30**, 4956-4972.e4.
