# Supplementary material for: The archaeal protein SepF is essential for cell division in Haloferax volcanii
Source: Nat Commun. 2021 Jun 8;12:3469. doi: 10.1038/s41467-021-23686-9 (PMC8187382; doi:10.1038/s41467-021-23686-9)
Supplement: Supplementary file 3 — Description of Additional Supplementary Files [file 41467_2021_23686_MOESM3_ESM.pdf]

Description of Additional Supplementary Files

File Name: Supplementary Movie 1
Description: Timelapse microscopy of H26 cells expressing SepF-GFP. Scale bar 4 µm.

File Name: Supplementary Movie 2
Description: Timelapse microscopy of HTQ239 cells during SepF depletion. Scale bar 4 µm.
