# Supplementary material for: The archaeal protein SepF is essential for cell division in Haloferax volcanii
Source: Nat Commun. 2021 Jun 8;12:3469. doi: 10.1038/s41467-021-23686-9 (PMC8187382; doi:10.1038/s41467-021-23686-9)
Supplement: Supplementary file 6 — Reporting Summary [file 41467_2021_23686_MOESM6_ESM.pdf]

nature research

Corresponding author(s): Sonja-Verena Albers

Last updated by author(s): Apr 26, 2021

Reporting Summary

Nature Research wishes to improve the reproducibility of the work that we publish. This form provides structure for consistency and transparency in reporting. For further information on Nature Research policies, see our Editorial Policies and the Editorial Policy Checklist.

Statistics

For all statistical analyses, confirm that the following items are present in the figure legend, table legend, main text, or Methods section.

n/a	Confirmed	
☐	☒	The exact sample size (n) for each experimental group/condition, given as a discrete number and unit of measurement
☐	☒	A statement on whether measurements were taken from distinct samples or whether the same sample was measured repeatedly
☐	☒	The statistical test(s) used AND whether they are one- or two-sided<br>*Only common tests should be described solely by name; describe more complex techniques in the Methods section.*
☒	☐	A description of all covariates tested
☒	☐	A description of any assumptions or corrections, such as tests of normality and adjustment for multiple comparisons
☐	☒	A full description of the statistical parameters including central tendency (e.g. means) or other basic estimates (e.g. regression coefficient) AND variation (e.g. standard deviation) or associated estimates of uncertainty (e.g. confidence intervals)
☐	☒	For null hypothesis testing, the test statistic (e.g. F, t, r) with confidence intervals, effect sizes, degrees of freedom and P value noted<br>*Give P values as exact values whenever suitable.*
☒	☐	For Bayesian analysis, information on the choice of priors and Markov chain Monte Carlo settings
☒	☐	For hierarchical and complex designs, identification of the appropriate level for tests and full reporting of outcomes
☒	☐	Estimates of effect sizes (e.g. Cohen's d, Pearson's r), indicating how they were calculated

Our web collection on statistics for biologists contains articles on many of the points above.

Software and code

Policy information about availability of computer code

Data collection	VisiView 4.5.0.6. was used to acquire microscopy pictures; CGQuant 7.4 was used to acquire growth data; Protein alignment was done using Jalview Version 2.11.0 with MUSCLE (default settings); PurityChrom® 5.09.036 from Knauer was used for protein purification and size exclusion chromatography
Data analysis	Fiji (Version 1.51) with MicrobeJ plug-in (Version5.13l) was used for analysis of microscopy Data and partially generation of graphs; Microsoft Excel 2013 and Graphpad Prism (Version 6.07) was used for growthcurves, Microscopy Data (co-localization, cell size analysis) and protein purification / size exclusion chromatography profiles; CorelDraw X5 was used to make the model

For manuscripts utilizing custom algorithms or software that are central to the research but not yet described in published literature, software must be made available to editors and reviewers. We strongly encourage code deposition in a community repository (e.g. GitHub). See the Nature Research guidelines for submitting code & software for further information.

Data

Policy information about availability of data

All manuscripts must include a data availability statement. This statement should provide the following information, where applicable:
- Accession codes, unique identifiers, or web links for publicly available datasets
- A list of figures that have associated raw data
- A description of any restrictions on data availability

The data that support the findings of this study are available from the corresponding author upon request. Source data are provided with this paper.

Field-specific reporting

Please select the one below that is the best fit for your research. If you are not sure, read the appropriate sections before making your selection.

☒ Life sciences ☐ Behavioural & social sciences ☐ Ecological, evolutionary & environmental sciences

For a reference copy of the document with all sections, see nature.com/documents/nr-reporting-summary-flat.pdf

Life sciences study design

All studies must disclose on these points even when the disclosure is negative.

Sample size	No statistical methods were used to predetermine sample size. Sample sizes were chosen based on historical data.
Data exclusions	In general only every tenth data point of the measured growth curve was plotted.<br>Growth curve Figure 4a: The last 14h were excluded as the cells were already in stationary phase. Figure 7: cells with no FtsZ rings were excluded from analysis<br>Microscopy Figure S8: at t = 24 cells that were longer than 32 um were excluded from the Demograph
Replication	Each experiment was at least performed in three biological replicates. All replication attempts were successful.
Randomization	Respective strains for different experiments were selected randomly for inoculation from plate. They were grown under similar conditions and were therefore equivalent at the start of experiments. Their differences observed can be attributed to the genotype or treatment under testing conditions.
Blinding	No blinding was performed as this was not necessary for our study. Strains were grown under similar conditions and were therefore equivalent at the start of experiments. Their differences observed can be attributed to the genotype or treatment under testing conditions.

Reporting for specific materials, systems and methods

We require information from authors about some types of materials, experimental systems and methods used in many studies. Here, indicate whether each material, system or method listed is relevant to your study. If you are not sure if a list item applies to your research, read the appropriate section before selecting a response.

Materials & experimental systems

n/a	Involved in the study	
☐	☒	Antibodies
☒	☐	Eukaryotic cell lines
☒	☐	Palaeontology and archaeology
☒	☐	Animals and other organisms
☒	☐	Human research participants
☒	☐	Clinical data
☒	☐	Dual use research of concern

Methods

n/a	Involved in the study	
☒	☐	ChIP-seq
☒	☐	Flow cytometry
☒	☐	MRI-based neuroimaging

Antibodies

Antibodies used	1. Anti-FtsZ1 antibody produced in rabbit (1:1000 dilution) were obtained from Iain Duggins Lab (Liao et al, biorvix, 2020)<br>2. Anti-FtsZ2 antibody produced in rabbit (1:1000 dilution) were obtained from Iain Duggins Lab (Liao et al, biorvix, 2020)<br>3. Anti-HA antibody produced in rabbit (1:10000 dilution) were obtained from Sigma-Aldrich (H6908)<br>4. Secondary Anti-rabbit HRP coupled antibody produced in goat (1:10000 dilution) provided by Invitrogen (65-6120)<br>5. Anti-His antibody (1:10000 dilution) dilution provided by Abcam (ab184607)
Validation	Validation for the Anti-HA, Anti-rabbit HRP and Anti-His antibody was based on technical data sheets provided by the manufacturers. FtsZ1 and 2 antibodies were validated before by Yiao et al. (Nat Microbiol (2021). https://doi.org/10.1038/s41564-021-00894-z). Additionally, as a control for the FtsZ1 and FtsZ2 antibodies, purified protein of FtsZ1 and FtsZ2 was loaded on the SDS gels to distinquis unspecific from specific signals.
